# Mechanical ventilation in dogs and cats with tick paralysis

**DOI:** 10.3389/fvets.2023.1071191

**Published:** 2023-04-06

**Authors:** Tess O'Keeffe, Rebekah E. Donaldson

**Affiliations:** Department of Critical Care, Queensland Veterinary Specialists, Brisbane, QLD, Australia

**Keywords:** positive-pressure ventilation, lower motor neuron disease, respiratory failure, hypoventilation, *Ixodes holocylus*

## Abstract

Respiratory failure from tick paralysis (TP) is an important cause of mortality in cats and dogs in Australia, occurring from a combination of respiratory muscle paralysis, upper respiratory tract obstruction and pulmonary disease. Patients may require positive-pressure ventilation in management of any combination of hypoxemia, hypoventilation or respiratory fatigue, but may also require airway management due to laryngeal paralysis. No single ventilation strategy is recommended due to the heterogenous disease presentations. Lung protective ventilation should be used in patients with pulmonary disease. Due to local and systemic effects of TP, patients are at higher risk of complications such as aspiration pneumonia and corneal ulceration and may have additional intravenous fluid and nutritional considerations. Treatment with hyperimmune serum is associated with improved outcomes. Prognosis is considered good with documented survival to discharge (STD) of 52.6–77% for animals with TP ventilated with lung disease and 90.5% for animals without lung disease. Median reported duration of ventilation for TP ranges from 23 to 48 h (range 3 h−10 days). The severity of individual neuromuscular signs and the presence of associated conditions such as aspiration pneumonia and laryngeal paralysis may necessitate longer periods of mechanical ventilation. This review aims to summarize the current recommendations regarding indications, management and prognosis of cats and dogs undergoing MV for TP and to identify areas for future research.

## 1. Introduction

Tick paralysis (TP) is a cause of morbidity and mortality in dogs and cats, with >10,000 pets estimated to present to veterinary clinics for treatment annually (1). Clinical disease is most common in animals that reside in known geographical tick regions but can occur in patients with a history of travel to or from these areas (1).

Within Australia, TP is caused primarily by the release of toxins from the salivary glands of adult female *Ixodes holocyclus* ticks (2), although there are reports of clinical disease in animals infested with a morphologically similar species *Ixodes cornuatus* (3, 4). In the United States of America, *Dermacentor* species cause paralysis that resolves with identification and removal of the tick (5).

The toxin prevents the presynaptic release of acetylcholine at the neuromuscular junction leading to a clinical syndrome characterized by ascending flaccid paralysis (6). Treatment involves administration of hyperimmune serum and supportive care until clinical signs resolve (7). Complete recovery from TP is possible but mortality rates of 2–5% (8, 9) in treated dogs and cats persist (7, 8). Death occurs due to respiratory failure (8–10).

A retrospective analysis of 2077 cats with TP demonstrated that MV was recommended in 6% of presenting cases (11). The proportion of dogs with TP that develop an indication for MV is currently unknown.

Mechanical ventilation (MV) is an important intervention in cats and dogs severely affected by TP. The objective of this review is to summarize the current recommendations regarding indications, management and prognosis of cats and dogs undergoing MV for TP and to identify areas for future research.

## 2. Pathophysiology and management of tick paralysis

The species of tick associated with paralysis in Australia are *I. holocyclus* and *I. cornuatus*, with each occupying a distinct geographical location. *Ixodes holocyclus* populates the coastline of Queensland and New South Wales with a small number of cases documented in Eastern Victoria (11, 12) and is the more significant to clinical disease. *Ixodes cornuatus* is more southernly distributed in Victoria and Tasmania (4, 12). Affected animals are presented to veterinary clinics year-round, although a distinct seasonal pattern exists, with a peak in spring and summer, coinciding with large numbers of adult females (11, 12).

Holocyclotoxin1 is a 5–6 kDa molecule produced in the salivary glands of the tick. Peak production occurs at day 3–4 following attachment and coincides with the development of clinical signs (2, 13). Holocyclotoxin1 (14) binds to the presynaptic neuromuscular junction, preventing the release of acetylcholine (6). The resulting clinical syndrome is primarily an ascending flaccid motor paralysis with progressive alteration in respiratory mechanics leading ultimately to respiratory failure in the untreated animal (10). Additional clinical signs may include dysphonia, vomiting, regurgitation (10), megaesophagus (15), bladder dysfunction (10), signs of left-sided congestive heart failure (16) and focal neurological deficits, including anisocoria, absent palpebral reflexes, asymmetrical facial paralysis, and laryngeal paralysis (14).

Conventional grading systems exist to define severity of clinical signs and are widely used in clinical practice (Table 1) (1, 17). Patients are assigned a gait and respiratory score at time of diagnosis. Increasing respiratory scores in dogs and increasing gait scores in cats are predictive of mortality (1, 17).

**Table 1:** Respiratory and gait scoring system for tick paralysis (17).

**Gait score**	
1	Mild weakness
2	Can stand but not walk
3	Cannot stand but can right itself and maintain sternal recumbency
4	Unable to right itself, cannot maintain sternal recumbency
**Respiratory score**	
A	Normal
B	Mild: increased respiratory rate and effort
C	Moderate: any respiratory distress or dyspnea, restrictive breathing pattern, coughing, gagging, or retching
D	Severe: severe dyspnea, cyanosis, progressive reduction in respiratory rate, open-mouth breathing

Proposed mechanisms of respiratory compromise include respiratory muscle paralysis in isolation or combination with upper respiratory tract (URT) obstruction (18, 19), central respiratory depression (20, 21), cardiopulmonary disease (16) and aspiration pneumonia (8, 9).

A study investigating lung histopathology in dogs euthanized for TP demonstrated pulmonary parenchymal changes in all samples submitted (*n* = 25) (8). Severe inflammation consistent with bronchopneumonia was present in 60% of samples, with the remainder showing evidence of pulmonary congestion and oedema (8). Another study examining lung histopathology of dogs and cats with fatal TP also found evidence of bronchopneumonia in 48% (11/23) of dogs but only 18% (2/11) of cats (9).

The primary cause of bronchopneumonia in animals with TP is believed to be aspiration (8, 9). The cause of pulmonary edema (PE) is less clear and may be multi-factorial (8, 9, 16).

A study of five dogs with TP demonstrated that the protein concentration of edema fluid was less than half the serum protein concentration. The authors proposed these findings were consistent with hydrostatic PE secondary to toxin-related cardiac dysfunction (16). Another study of post-mortem lung histopathology identified 10/25 dogs had pulmonary parenchymal disease without evidence of bronchopneumonia (8). Hydrostatic pulmonary edema was deemed the most likely mechanism in the absence of fibrin deposition, hyaline membranes, and alveolar cellular infiltrate in these dogs (8). These findings are insensitive and non-specific for diagnosing the etiopathogenesis of pulmonary oedema (8).

A combined retrospective and prospective study of lung histopathology in fatal TP demonstrated evidence of high protein oedema and fibrin exudation in 100% (23/23) and 69.6% (16/23) of dogs and 100% (11/11) and 90.7% (10/11) cats, respectively (9). Toxin-induced acute lung injury or inflammatory reactions were suggested as possible mechanisms (9). Negative pressure pulmonary oedema secondary to upper respiratory tract obstruction is also recognized in humans and dogs with laryngeal paralysis (22, 23). Whether laryngeal paralysis plays a role in the development of PE in TP is unknown.

Vomiting and regurgitation are common presenting signs of tick paralysis in dogs and cats (10, 24). Regurgitation may occur secondary to megaesophagus and is a major contributor to aspiration pneumonia in dogs with TP (15). Esophageal motility disorders are uncommon in cats (25) due to differences in the smooth muscle content and innervation of the distal third of the esophageal body compared to dogs (26). The literature suggests bronchopneumonia is less common in cats with TP and supports hypoventilation as the primary cause of respiratory failure in this species (9).

Acute management of TP involves intravenous administration of tick anti-serum (TAS), clipping of the coat and topical application of a rapid kill acaricide to locate and remove any remaining ticks (27, 28). Serial tick searches should be performed multiple times a day to facilitate prompt removal of any ticks not killed by the acaricide (27, 29). It is unclear if removal of additional ticks after treatment necessitates repeat administration of TAS. Innate immunity and the circulating half-life of TAS may influence the need for additional doses; further research is required in this area. One prospective survey reported 20% lower survival in dogs receiving multiple TAS doses for a single exposure compared to those receiving a single dose (1). The rationale for additional doses was not reported, so it is unclear if clinical deterioration or illness severity prompted additional TAS administration as opposed to the discovery of additional ticks.

Supportive care should be tailored to individual patient needs and may consist of intravenous fluid therapy, supplemental oxygen, sedation, medicated ocular therapies, physiotherapy, bladder management, prokinetics and proton pump inhibitors (27, 28, 30). Broad-spectrum parenteral antibiotic therapy is indicated in patients with a confirmed or suspected diagnosis of aspiration pneumonia (28). Appropriate antibiotic selection is associated with survival to discharge (odds ratio 45.8, 95% CI 1.98–14,808) (31). Lower airway culture sampled by endotracheal wash or bronchoalveolar lavage is recommended and antibiotic de-escalation driven by these results.

In depth discussion of the management of animals with TP but without respiratory failure is beyond the scope of this review and the author directs the reader's attention to the recent clinical guidelines provided by an expert advisory panel (28).

## 3. Indications for mechanical ventilation in animals with tick paralysis

Animals with TP are at risk of respiratory failure from hypoventilation, hypoxemia, and unsustainable respiratory effort (20, 21). MV is indicated when severe hypoventilation is demonstrated by significant hypercapnia [arterial partial pressure carbon dioxide (PaCO_2_) >60 mmHg] despite therapy or when severe hypoxemia [arterial partial pressure oxygen (PaO_2_) <60 mmHg or peripheral oxygen saturation (SpO_2_) < 90%] persists with supplemental oxygen. Unsustainable respiratory effort with concern for impending respiratory arrest is another important indication and is a subjective assessment based on clinical judgement (32).

A retrospective multi-center study investigating the indications of MV in dogs and cats with TP found that 38.9% (21/54) of dogs were ventilated for hypoxemia, 33.3% (18/54) for hypercapnia, 18.5% (10/54) for unsustainable breathing effort and 7.4% (4/54) for fatigue resulting in respiratory arrest (30). Cats were most frequently ventilated for hypoventilation [71.4% (5/7)], with 14.3% (1/7) ventilated due to hypoxemia (30). This is reflective of the low histopathological incidence of pulmonary parenchymal disease in this species (9).

Arterial blood gas analysis remains the gold standard assessment of oxygenation; however, it is technically challenging and not always practical in a conscious, dyspneic patient (33). A prospective observational study demonstrated pulse oximetry to be an insensitive measure of hypoxemia in the conscious dog (34). The venous partial pressure of carbon dioxide (PvCO_2_) and the end tidal CO_2_ (ETCO_2_) obtained *via* capnography are accepted surrogate markers for PaCO_2_ in the absence of increased physiological dead space, returning values 2–10 mmHg higher and lower than PaCO_2_, respectively (35, 36).

Early intubation of dyspneic patients protects the airway from oropharyngeal and gastric secretions (37), relieves possible URT obstruction (38) and allows for more accurate assessment of respiratory mechanics, either *via* blood gas analysis (36) or capnography (35) and pulse oximetry (34). Intervention should not be withheld based on an inability to document absolute indication for MV and should be performed if the clinician deems appropriate.

## 4. Management of the ventilated patient

### 4.1. Airway management

Endotracheal intubation and general anesthesia are required to maintain MV in most veterinary patients (39). A recent retrospective study suggests TP dogs and cats can require several days of MV (median 4 days, range 1–10 days) (31), so care must be taken to mitigate complications associated with prolonged endotracheal (ET) intubation, which include pressure necrosis (40), obstruction or dislodgement of the tube and introduction of pathogens into the airway, with subsequent development of ventilator-associated pneumonia (VAP) (41, 42). Incidence of VAP in dogs and cats with TP is reported as 3.8% (1/26) in a retrospective analysis of patients with positive airway cultures (31). Care must be taken to ensure that the ET tube (ETT) is placed in a sterile manner and secured with non-porous material that does not promote formation of biofilm (43), such as sterile fluid extension lines. Stringent attention should be paid to hand hygiene and examination gloves worn for all subsequent management of the airway.

Cuff-induced tracheal injury, encompassing tracheal mucosal irritation, rupture, pressure necrosis and post-intubation stenosis, are important complications of ET intubation in cats and dogs (44–46). Cuff pressures should be high enough to provide a seal and prevent aspiration of oral secretions around the cuff without compromising tracheal blood flow (47). Pressure ranges of 20–30 cmH_2_O are standard and have been extrapolated from human data, with the goal to remain below tracheal capillary perfusion pressure, which ranges from 30–43.5 cmH_2_O (47). The use of commercial syringe devices designed for ETT cuff inflation is recommended, as they have been shown to be superior to the minimum occlusive volume technique or palpation of the pilot balloon for estimating cuff pressure (47). Performance of tube care (oropharyngeal suctioning and measurement of cuff pressure) every 4 h is recommended to reduce the likelihood of tracheal pressure necrosis (48).

Aspiration pneumonia is an important consequence of TP (8, 9, 30), with megaesophagus (15) and laryngeal paralysis (19) predisposing this subset of patients to aspiration of oropharyngeal and gastric secretions. Bronchopneumonia has been demonstrated in lung samples of 48–60% of dogs and 18% of cats affected by the disease (8, 9). Furthermore, micro-aspiration of oral secretions is thought to be the first step in the pathogenesis of VAP in people (49). To reduce the incidence of VAP, the American Thoracic Society recommends cuff pressures >20 cmH_2_O, suctioning of the oropharynx to remove oral secretions above the cuff prior to tube repositioning and tube replacement only as required to manage obstruction by respiratory secretions (50).

Temporary tracheostomy (TT) and cricothyrotomy (CTT) tubes are recommended in animals with generalized LMN disease requiring long-term ventilation (51) or to facilitate weaning in animals with URT obstruction. Their use has been described in cats and dogs with TP (18, 52). Benefits of a surgical airway include tracking of neurological progress, reduction of costs associated with anesthetic drugs, shorter ventilation times and improved weaning rates for animals with laryngeal paralysis secondary to TP (52). Common reported complications of TT include obstruction and dislodgement of the tube, as well as aspiration pneumonia (53).

When considering a patient for a surgical airway, a clinician should weigh the risk and costs of TT/CTT against the expected time and cost of continued MV. A surgical airway in TP patients is indicated to facilitate weaning in animals that are expected to fail extubation due to ongoing laryngeal paralysis (19, 52, 54). As focal neurological deficits can persist for days to weeks after resolution of generalized paralysis (14), experts recommend TT to improve weaning rates in this subset of patients (28, 52).

One case report describes the successful use of a cricothyrotomy tube in a dog undergoing MV for TP and aspiration pneumonia that failed weaning due to persistent laryngeal paralysis (18). This was the first reported use of a CTT in clinical practice and the author noted a less technically challenging procedure, ease of tube replacement due to an anatomically robust stoma and the need for only one stay suture as potential benefits over the more traditional TT (18). Studies comparing outcomes and complication rates of each procedure are required before recommendations can be made for one technique over another.

Animals with TP that require intubation for URT obstruction or airway protection should be considered for MV as apparatus dead space (55) and the respiratory depressive effects of sedatives (32) may contribute to hypoventilation, atelectasis, and work of breathing (52). ICU ventilators provide real time feedback on respiratory mechanics, allow the operator to set fraction of inspired oxygen (FiO_2_) and employ lung protective strategies by administration of set tidal volumes, inspiratory pressures, and positive end expiratory pressure (PEEP) (55). If an ICU ventilator is not available, anesthetic ventilators or manual ventilation can be used but lack the ability to titrate oxygen concentration and may increase the likelihood of ventilator induced lung injury (VILI) from barotrauma or volutrauma (32). Intubated, non-ventilated patients also are at risk of atelectasis due to an inability to provide PEEP (56). In animals that are intubated but not ventilated, intratracheal oxygen may be titrated *via* a sterile feeding tube down the ETT with flow rate adjustments to meet PaO_2_ targets (53). Humidified air can be supplied to intubated, non-ventilated patients *via* intermittent nebulization, however, they remain at increased risk of VAP from an open system (57).

Dogs and cats mechanically ventilated for pulmonary parenchymal disease have higher mortality rates (23–47.4%) than those receiving positive pressure ventilation (PPV) for ventilatory failure (9.5%) (30, 31). With the high incidence of bronchopneumonia in dogs with TP (8, 9), future research should evaluate whether early and aggressive airway management would improve survival rates for these animals.

### 4.2. Anesthesia

General anesthesia is typically required to facilitate invasive mechanical ventilation. Total intravenous anesthesia (TIVA) is preferred as inhalant anesthesia (32) may cause greater cardiopulmonary depression, suppression of hypoxic pulmonary vasoconstriction (58, 59), and pose greater exposure risks for staff (32). When selecting agents for TIVA, a clinician must consider the hemodynamic effects, duration of action and mode of elimination of each medication and how it may be of complement or detriment to the patient's physiologic state. Typically, a multi-agent balanced anesthetic approach allows lower dose rates of each drug, in turn minimizing adverse effects (60). Patient-ventilator asynchrony has been described in a patient receiving propofol 0.2–0.4 mg/kg/min as the sole anesthetic agent (61). In this case report, a progressive increase in the required propofol dose coincided with the return of strong ventilatory efforts seen with the gradual resolution of the patient's polyridiculoneuritis (PRN) (61). Given the clinical similarities between PRN and tick paralysis, it is logical to theorize that an increase in propofol dose over time would be expected in animals ventilated for tick paralysis as the underlying disease process resolves.

Combinations of opioids, benzodiazepines, and anesthetic agents, including propofol and ketamine, have been described in small animals with good success (62, 63). A standardized anesthetic protocol using constant rate infusions (CRIs) of propofol (0.05–0.4 mg/kg/min), butorphanol (0.1–0.4 mg/kg/h) and midazolam (0.1–0.5 mg/kg/h) was described in dogs and cats undergoing mechanical ventilation for tick paralysis, with no reported anesthetic complications. No similar studies exist to compare protocols, but the high survival to discharge rate (75% when financial euthanasia was excluded) suggests appropriateness of this protocol (30). The authors' institution uses fentanyl CRI in preference to butorphanol as it is easily titratable and as a pure-mu opioid agonist is more amenable to reversal. Sufentanil (0.5 mcg/kg/h) has been used in a case of PRN successfully weaned from mechanical ventilation (51). Dexmedetomidine (0.5 mcg/kg/h) was used in the same protocol with maintenance of hemodynamic stability and facilitated 50% reduction in propofol dose and rapid extubation (51). Medetomidine CRIs have been used at the authors' institution with anecdotal success to facilitate weaning in patients with tick paralysis.

While midazolam is frequently used in TIVA protocols, withdrawal syndromes are associated with long-term benzodiazepine use in humans and are an important consideration for patients receiving long term mechanical ventilation. Signs of withdrawal develop within 48 h of discontinuation of therapy and have been associated with agitation, muscle twitching, disorientation, and seizures, with an overall higher prevalence in children (64, 65). Midazolam withdrawal was suspected in a case series of generalized seizures in three mechanically ventilated puppies (2–5 months old), based on exclusion of systemic disease with biochemistry, timing, resolution of signs with reintroduction and subsequent weaning of benzodiazepines over 24–48 h and an absence of seizures at long-term follow-up (66). In this case series, the dose of midazolam used was high (0.3–1.5 mg/kg/h) in 2/3 cases (66), exceeding the rate recommended for management of TP (0.1–0.3 mg/kg/h) (28). Causation cannot be proven in this study with limitations including the absence of intracranial work-up for all patients and the presence of severe hypoxemia (67) and atracurium administration (68) as cofounding variables. Midazolam is also associated with respiratory muscle paresis in humans (69). The above highlights the importance of multi-agent balanced anesthesia in reducing the side-effect profile of individual agents due to the dose reduction afforded by the synergy of the medications chosen (60).

Comparative studies are needed to determine whether the functional neuromuscular blockade caused by holocyclotoxin allows for lower anesthetic doses in patients with TP. As naturally occurring lower motor neuron paralysis is present, neuromuscular blocking agents may not provide additional ventilatory control and their use should be carefully considered with appropriate troubleshooting for other causes of patient-ventilator asynchrony.

### 4.3. Ventilator settings

The goal of mechanical ventilation is to maintain appropriate blood gas parameters with the least aggressive ventilator settings. Ideally, the clinician should aim for a partial pressure of arterial oxygen (PaO_2_) between 80 and 120 mmHg and a partial pressure of arterial carbon dioxide (PaCO_2_) between 35 and 50 mmHg (39), although targets of PaO_2_ > 60 mmHg, SpO_2_ > 90% and PaCO_2_ <60 mmHg, have been used successfully in animals with tick paralysis (30).

Optimal ventilator settings in cats and dogs with naturally occurring tick paralysis, neuromuscular, or pulmonary disease are unknown. Pressure-control, volume-control and synchronized intermittent mandatory ventilation (SIMV) modes of ventilation have been described for use in TP (18, 30), however, no literature exists (human or veterinary) that compares their use or outcomes for this patient subset. The general recommendations for initial settings in patients with pulmonary and non-pulmonary disease are described elsewhere (39) and are predominantly extrapolated from small studies in healthy animals and available human clinical trials. A single case report detailing the successful management of a dog with TP and associated aspiration pneumonia and laryngeal paralysis described volume-controlled mandatory ventilation, FiO_2_ 1.0, Tidal volume (Vte) 8 ml/kg and PEEP 5 cmH_2_O as initial settings (18). While guidelines are a useful starting point, settings must ultimately be tailored to the individual and optimization can only be determined with trial and error (39, 70).

Patients with pulmonary parenchymal disease have reduced lung compliance and may require more aggressive ventilator settings than patients with ventilatory failure alone (32). PPV is not a benign process and the effects of VILI in pathological and pre-injured lung have been recognized in human medicine (71, 72).

Plateau pressures > 30 cmH_2_O, tidal volumes > 10 ml/kg and cyclic alveolar opening and closing are the main determinants of VILI in humans (71–73). Patients with pulmonary parenchymal disease are more susceptible to VILI due to heterogenous compliance of lung units predisposing to alveolar overdistension, biotrauma secondary to inflammation and decreased surfactant from alveolar flooding necessitating higher driving pressures (71, 72).

A small experimental study of anesthetized healthy adult cats receiving pressure-controlled mandatory ventilation with inspiratory pressures (Pi) of 5 and 7 cmH_2_O and 0 cmH_2_O PEEP generated a Vte of 6–10 ml/kg with minimal overinflation as assessed by computed tomography (CT) (74). At 15 cmH_2_O Pi, CT identified a significant increase in overinflated lung occurred (74). These findings suggest that initial inspiratory pressures of 5–7 cmH_2_O are appropriate in dogs and cats without pulmonary disease.

Lung protective ventilation strategies have been demonstrated to decrease mortality in critically ill, mechanically ventilated humans with acute respiratory distress syndrome (ARDS) (75). While most literature supports a benefit in humans with, or at risk of, ARDS, there is evidence to suggest a wider population may benefit from these strategies given the risk of VILI in all patients receiving PPV, (76). Current human guidelines recommend the use of low tidal volumes (typically 6–8 ml/kg), to limit alveolar overdistension, and moderate-high PEEP (5–12 cm H_2_O) to promote alveolar recruitment and reduce sheering injury (56, 77). Permissive hypercapnia refers to the tolerance for respiratory acidosis in these patients provided they continue to oxygenate effectively and remain cardiovascularly stable (76). Additionally, the sympathomimetic effect (increased HR and BP) of increasing PaCO_2_ can be beneficial in moderating the cardiovascular depressive effect of high PEEP (78). Pilot studies evaluating lung protective strategies in dogs have demonstrated the techniques are well-tolerated in healthy animals (79) and there is some indication of reduced inflammation in experimentally induced models of acute lung injury (80, 81).

A randomized control trial of 40 dogs without pulmonary disease undergoing CT were anesthetized and randomized to four groups: Vte 8 ml/kg with or without 5 cm H_2_0 PEEP and Vte 15 ml/kg with or without 5 cm H_2_O PEEP (74). Atelectic, overinflated and normally aerated lung units did not differ between groups at end inspiration, but at end expiration dogs receiving Vte 15 ml/kg with PEEP had significantly more normally aerated lung and significantly less poorly- or non-inflated lung than the other groups (74). Oxygenation was not different between groups (74). These findings suggest that the application of PEEP may be more important to prevent lung heterogeneity than Vte specifically, supporting the safety of lower Vte in dogs in which lung protective ventilation is indicated.

Patients with reduced pulmonary compliance and persistent hypoxemia secondary to ventilation-perfusion (VQ) mismatch may benefit from alveolar recruitment maneuvers (ARMs) (76, 82, 83). These strategies aim to open (recruit) alveoli by applying a short-term increase in transpulmonary pressure and to subsequently prevent alveolar collapse (decruitment) by implementation of moderate-high PEEP following the maneuver (84). Current recommendations in humans with uninjured lungs are that ARMs should be performed with PEEP titration at low tidal volume when O_2_ saturation is below 92% (85). Various methods applied to anesthetized dogs and cats with healthy lungs have shown that ARMs increase pulmonary compliance and may increase or have no effect on oxygenation (74, 83, 84, 86). Transient alveolar overdistention (84), increased plateau pressures and a decrease in mean arterial pressure (MAP) have been demonstrated during maneuvers (74, 82–84) and the risk of volutrauma, barotrauma and hemodynamic compromise should be recognized in these patients. A prospective clinical study demonstrated evidence of alveolar overdistension in healthy dogs using a stepwise increasing/decreasing PEEP recruitment maneuver at tidal volumes of just 7 ml/kg (84). A recent systematic review on the use of ARMs in people with severe pulmonary disease, found that transient increases in oxygenation were of uncertain benefit in patients with ALI, recommending maneuvers be considered for individuals with life-threatening hypoxemia rather than on a routine basis (87). To the authors' knowledge there is no published literature on the use of ARMs in veterinary patients with diseased lungs. Further research is required before recommendations can be made for their use in this patient subset.

An invasive, mandatory mode of ventilation is recommended initially to provide airway protection and relieve work of breathing in patients experiencing neuromuscular paralysis and potentially respiratory fatigue, respectively (61). Most existing veterinary literature supports the initial use of a continuous-mandatory mode (assist-control) (30, 68, 88). While successful MV with synchronized intermittent mandatory ventilation (SIMV) has been described in tick patients, multi-center human trials demonstrated a longer time to extubation in patients on SIMV over other modes of ventilation (89).

Continuous spontaneous ventilation modes (such as pressure support ventilation) are most suited to patients with reduced ventilatory capacity and normal respiratory drive. This mode is unlikely to be appropriate in the initial phases but should be considered in patients recovering from respiratory paralysis (such as those with TP) and when assessing suitability for weaning (61, 90).

Following initiation of ventilation, the patient should be monitored continuously, and the suitability of settings assessed by ensuring respiratory mechanics and arterial blood gas parameters are within recommended targets (39). All patients should commence ventilation on a fraction of inspired oxygen (FiO_2_) of 1.0. Once the patient is stable a gradual reduction in FiO_2_ is typically the first adjustment made, with the goal of reducing inspired oxygen below the threshold for toxicity (<0.6) (91). Following this, adjustment of other settings can proceed and will depend on the mode of ventilation chosen by the operator.

Trigger variables should be set to facilitate spontaneous respiratory efforts. Unlike mandatory breaths, spontaneous breaths can be triggered by a detected change in flow or pressure. The trigger sensitivity should be set so that genuine respiratory efforts can be recognized by the machine to increase patient comfort with care not to be set so low that patient movement or routine patient care triggers breaths artefactually (61). While the trigger can be adjusted to prevent spontaneous efforts, this may mask patient-ventilator asynchrony and delay appropriate troubleshooting. Patients suffering lower motor neuron disease may be unable to initiate strong spontaneous breaths and are likely to benefit from low trigger variables (61). Text recommendations for initial MV trigger variables in dogs and cats are a flow rate of 1–2 L/min or pressure of 1–2 cmH_2_O (32). To the authors' knowledge, no literature exists detailing optimal trigger variables in dogs or cats with TP. A case report of a puppy receiving MV for PRN, a morphologically similar disease process to TP, reported an initial flow trigger of 0.5 L/min. As the patient regained muscular strength, the flow trigger needed to be increased to 1 L/min (61).

The cycle variable is the parameter terminating inspiration (92, 93). Time is the most common cycle variable used and may be determined by the inspiratory time or inspiratory/expiratory (I:E) ratio set by the operator (55). These variables may be manipulated to minimize hypercapnia when lung protective ventilation strategies are used; this subset of patients is expected to be relatively tolerant of permissive hypercapnia. No veterinary thresholds for permissive hypercapnia exist, so it is reasonable to apply limits of pH 7.2 and PaCO_2_ 80 mm Hg as defined in the human literature (94).

The use of pressure-control and volume-control ventilation has been described in patients requiring MV for tick paralysis and other causes (18, 30, 42, 61, 95). Pressure-control ventilation has been postulated to reduce patient work of breathing and improve patient comfort (96), however, a recent systematic review did not find any difference in outcomes between the mode chosen (97). The authors routinely use pressure-control ventilation, but there is insufficient evidence to recommend one mode over another.

It seems reasonable that the neuromuscular blockade effected by holocyclotoxin would reduce patient-ventilator asynchrony (which does not appear to be a common complication) (30, 88) and negate the need for pharmacological neuromuscular blockade. Further research is required before specific recommendations on ventilator settings in tick paralysis patients can be made.

### 4.4. Patient monitoring

Successful management of the ventilated patient requires continuous monitoring and exemplary nursing care. At a minimum, electrocardiography (ECG), blood pressure, pulse oximetry and capnography should be continuously monitored, as spontaneous breathing cannot be reliably observed (32).

Although technically challenging, placement of an arterial catheter is recommended for real-time hemodynamic monitoring and ease of sampling for arterial blood gas analysis, as retrospective analysis of ventilated dogs demonstrated pulse oximetry to be an insensitive measure of hypoxemia (34). Multi-lumen central venous catheters or peripherally inserted central catheters can be effective at preserving peripheral vasculature when regular blood sampling is required, for administration of parenteral nutrition, irritant solutions or coadministration of medications that would otherwise be contraindicated due to risk of precipitation (32).

### 4.5. Nursing care

Continuous and exemplary nursing care is crucial for the successful management of patients undergoing MV (70, 98). Careful thermoregulation, physiotherapy, and rigorous eye, oral, airway and bladder care are necessary to minimize complications of PPV, which include corneal, and oral ulceration, VAP, patient-ventilator asynchrony, and decubital ulcers (48). It may be difficult to differentiate complications of MV from those of the neuromuscular paralysis caused by TP, and it is unclear in the literature if there is a compounding effect of the two.

ICU hospitalization (99) and general anesthesia (100) have been associated with decreased tear production in dogs. In addition to decreased tear production, chemosis may develop in patients immobilized for long periods and can contribute to lagophthalmos and exposure keratopathy (98). Corneal ulceration has been reported in 5–36% of patients undergoing MV for all causes (41, 42). Current recommendations are that ocular care be performed every 2 h and encompass gentle cleaning with saline-soaked swabs and administration of artificial tears (70, 98). In a population of healthy anesthetized dogs, no difference in the incidence of corneal ulceration was found when the use of artificial tears alone was compared to administration of artificial tears and closure of the eyelids with tape (100). Fluorescein stain should be administered daily to evaluate both the presence and/or progression of corneal ulcers (70). If corneal ulceration is identified, broad spectrum topical antibiotics and atropine should be administered to prevent infection and the development of synechiae, respectively (98). Temporary tarsorrhaphy has been described in one patient with progressive bilateral corneal ulcers where protracted MV was anticipated (41). The ocular outcome in this case was not reported and there is no literature that directly evaluates the superiority of temporary tarsorrhaphy over standard medical care. Temporary tarsorrhaphy may also prevent serial cranial nerve assessment in the recovering TP patient.

Oral lesions have been reported in 37–90.5% of dogs receiving orotracheal MV (41, 101). The most common lesions include erosions or ulceration of soft tissue structures subjected to continuous pressure from teeth, monitoring equipment, tube ties or mouth gags, as well as, swelling and ranulas of the tongue (41, 101). The oral cavity should be inspected for lesions and care performed at least 4–6 hourly and may be required more frequently in patients with copious oral secretions (98). Mouth gags and monitoring equipment should be removed, and the tube cuff pressure checked for appropriate inflation prior to gentle suctioning of the oropharynx (98, 101). The tongue, mucous membranes and oropharynx should then be wiped and flushed with 0.05% chlorhexidine solution before the oropharynx is again suctioned (28, 98). Tube ties should be loosened, and the knot repositioned prior to replacement of monitoring equipment and small mouth gags in a different position to previous (98). Subglottic secretion drainage has been associated with reduced rates of VAP in people (102). Patients should be preoxygenated with 100% O_2_ for 2 min prior to oral and ETT care.

Facial nerve paralysis (14), megaesophagus (15), laryngeal paralysis (19) and toxin-associated autonomic dysfunction (10) place animals with TP at risk of corneal ulceration (14), bronchopneumonia (8, 9) and overflow incontinence (10), respectively. To the authors' knowledge, no study exists comparing the incidence of such complications between animals mechanically ventilated for TP and those for other disease processes. Regardless, these patients have multiple known risk factors for ventilator-associated complications, and it is imperative that clinicians pay careful attention to these elements of the patient care plan.

### 4.6. Intravenous fluid therapy

Pulmonary oedema is a prominent feature in animals with TP, however the underlying mechanism for its development remains poorly understood (8–10, 16). A study of 46 dogs with TP demonstrated low protein pulmonary oedema due to reversible diastolic dysfunction causing left-sided congestive heart failure (16). However, a subsequent study of 80 dogs with TP and 31 healthy control dogs failed to show an increase in the biomarker cardiac troponin I, suggesting an absence of myocardiocyte injury in animals affected with the disease (103).

High protein alveolar fluid and fibrin deposition was identified in pulmonary histopathology of cats and dogs that died from TP without obvious evidence of bronchopneumonia. The authors postulated these changes were suggestive of increased permeability oedema and an acute inflammatory process or lung injury (9). Given these findings, one may hypothesize that a combination of increased hydrostatic pressure and altered permeability of the pulmonary vasculature contributes to the development of oedema in animals with TP.

The apparent need for judicious fluid therapy in these patients does not preclude its use entirely. Animals with TP are routinely kept nil per os (to reduce the possibility of aspiration secondary to megaesophagus, laryngeal paralysis and reduced gag reflex) until clinical signs resolve (15), placing them at risk of dehydration and hypovolemia. Furthermore, it is well-recognized that MV induces changes in the loading conditions of the cardiac ventricles and hemodynamic fluid balance of the body (104). Adverse effects associated with positive or negative fluid balance have the potential to increase morbidity in cats and dogs with TP (28). They may be avoided by the judicious monitoring and matching of patient inputs (medications, intravenous fluids, and nutrition) and outputs (urinary, fecal, gastric residual volumes and oral secretions) (105). Serial assessment of body weight is a quick, inexpensive, and sensitive measure for monitoring fluid shifts within the body and should be performed at least every 12 h (28). Current recommendations from an expert panel are maintenance rates of 2.5 ml/kg/h with replacement volume if clinical evidence of dehydration is >7.5% (28).

### 4.7. Nutrition

Nutritional support is recommended for patients in caloric deficit for >72 h (106). Malnutrition in mechanically ventilated patients may contribute to weakness of the respiratory muscles and difficulty weaning (107). Additionally, in critically ill humans, malnutrition has been associated with immune-impairment, increased risk of infection and high mortality rates (108), with consensus guidelines recommending appropriate caloric intake be a prominent component of therapy (109). The same principles are likely to hold true for small animal patients, although care must be taken when extrapolating from human data due to inherent differences between veterinary and human ICU populations. A recent audit on the provision of nutritional support to mechanically ventilated patients found that just 31% (18/58) of animals receiving PPV had caloric supplementation. Of the animals that did not receive nutrition, 22.5% (13/58) exceeded 72 h of absent caloric intake without obvious documented contraindication to both enteral and parenteral administration, highlighting the ongoing need for clear evidence-based guidelines in this space (110).

In mechanically ventilated patients, nutrition may be delivered enterally or parenterally (107). Provision of enteral nutrition (EN) is preferred as it allows for maintenance of gastrointestinal mucosal integrity, reduction of bacterial translocation and decreases the metabolic response to stress (111). Contraindications to EN include persistent severe hypotension, trauma or infection at the proposed feeding tube site, and the presence of vomiting, regurgitation, ileus or megaesophagus. Reported complications of EN include vomiting or regurgitation with subsequent aspiration, dislodgement of the tube, delivery of nutrition outside of the gastrointestinal tract and infections at the stoma site (110). Critically ill animals receiving MV are at risk of gastrointestinal and esophageal dysmotility (107). Additionally, megaesophagus is prominent features of TP (15) and a clinician should be conscious of the significant aspiration risk these cases present when considering an EN feeding plan.

For severely affected animals requiring long-term ventilation, PN may be required supplementary to or instead of EN, given contraindications for EN are prominent features of TP (15). Parenteral nutrition (PN) requires administration *via* a dedicated port in single or multi-lumen jugular or long-stay peripheral catheter and contraindications include inappropriate vascular access, uncontrolled diabetes mellitus and hypervolemia (110). PN is indicated in patients unable to achieve nutritional goals by EN due to gastrointestinal dysfunction. Reported complications include mechanical failure, sepsis, and inflammation at the site of vascular access (112).

Little information is available regarding nutritional support for animals with TP. Current recommendations from an expert panel are that patients remain nil per os until clinical signs of megaesophagus, laryngeal paralysis and reduced gag reflex have resolved (28). Given the purported benefits of early goal directed nutrition in critically ill ventilated humans (108) and the inability to accurately assess neurological progress in anesthetized patients, the nutritional recommendations for ventilated animals with TP may differ from that of conscious, cardiovascular, and respiratory stable patients (28).

EN *via* nasogastric (NG) tube is a minimally invasive and cost-effective (113) way to provide nutritional support to mechanically ventilated patients with TP. Due to the increased risk of regurgitation and aspiration in this population (30), patients with TP should only be considered for enteral nutrition if they are cardiovascularly stable, have no regurgitation and have gastric residual volume (GRV) <10 ml/kg (107). Animals meeting these criteria should be commenced on a low percentage of their daily recommended energy requirement (RER) and very gradually increased if tolerance is demonstrated by continually low GRV (114). Serial point of care ultrasound is an effective bedside test that can be implemented to assess gastric content and volume (115). During the weaning period, an NG tube can be utilized to empty gastric contents (116), reducing the risk of aspiration.

Nasoesophageal and esophagostomy tubes are contraindicated in patients with megaesophagus (15, 110) and cannot be utilized for gastric decompression (116). Due to the transient and reversible nature of tick paralysis, percutaneous gastrostomy tubes are not currently recommended (113).

The median time for MV in animals with TP is 23 h−4 days (30, 32, 40), suggesting enteral nutrition is likely to be required in these patients. The timing of realimentation must be considered against the potential risks of early nutritional support. Future research should evaluate outcomes and complications of nutritional support in animals with TP.

### 4.8. Weaning

To date, there has been no evaluation of predictive indices of weaning in veterinary medicine. The decision to wean is dependent on clinical judgement, invariably involving a degree of trial and error (90, 117). The primary disease process (42, 95, 118), duration of ventilation (95), disuse atrophy of the respiratory muscles (119, 120), ventilator-associated complications (41, 90), species (42, 95) and sedative protocols used (62, 63) will contribute to the success or failure of weaning.

Suitability for weaning may be considered when the patient is cardiovascularly stable, there is clinical improvement in the underlying disease process and the patient's ventilatory load and capacity are such that they can maintain respiratory mechanics and arterial blood gases within acceptable physiological ranges on minimal ventilator support (90, 121). Specifically, a patient should be able to maintain a PaO_2_ > 80 mmHg on FiO_2_ < 0.6 and PEEP < 5.0 cmH_2_O, and their ventilatory drive and capacity should be such that they are able to maintain a PaCO_2_ < 60 mmHg (90, 117). The use of checklists to assess suitability for weaning have been described in veterinary medicine and the interested reader is directed to a review on the topic (90). As discontinuation from MV can be a high-risk time for the patient, it is recommended that breathing trials are performed during periods of maximal staffing capacity to ensure close monitoring and adequate staff are on hand should complications arise (90).

Several methods of ventilator weaning have been described and compared in human clinical trials (89, 122, 123). Spontaneous breathing trials (SBTs), synchronized intermittent mandatory ventilation (SIMV) and pressure support ventilation are commonly used in both human and veterinary medicine (90, 117, 122, 124).

During a spontaneous breathing trial, the patient is removed from the ventilator and allowed to breathe oxygen enriched air through a Bain circuit (90). This method forces the patient to abruptly take over the work of breathing and the determination of readiness to wean is largely subjective due to the loss of machine monitoring. Alternatively, a patient can remain attached to the ventilator and a low level (2–5 cmH_2_O) of continuous positive airway pressure applied (117) to overcome the work of breathing associated with the dead space and resistance from the machine and circuit (125).

The SIMV mode of ventilation provides a fixed number of mandatory breaths, with the patient able to make spontaneous efforts at other times. During weaning, a clinician can slowly decrease the number of mandatory breaths delivered, gradually increasing the work of breathing to the patient (90, 117). It was originally thought that this gradual transfer of effort may be beneficial to patients with respiratory muscle fatigue; however, several multi-center human trials demonstrated a longer duration of weaning when this method is used over SBTs or pressure support modes (89, 125). There are currently no veterinary clinical trials comparing weaning modes and the efficacy of SIMV in cats and dogs remains unknown.

In pressure support mode, the patient is allowed to initiate and cycle breaths through the ventilator circuit and the machine augments the breath with an inspiratory pressure set by the operator. The operator is gradually able to decrease the amount of pressure support over time and the patient maintains control of respiratory rate, tidal volume, and inspiratory flow (90, 117). One clinical trial demonstrated this method of weaning to be superior to a SBT and SIMV as a means of weaning (123). Pressure support mode weaning is the method predominantly used at the authors' institution with anecdotal success in TP patients.

Cats and dogs with TP are likely to have a degree of respiratory muscle weakness contributing to respiratory failure (10). Additionally, serial neurological assessment can prove challenging in the mechanically ventilated anesthetized individual, making it challenging to determine resolution of the primary disease process. Consequently, a mode which allows demonstration of appropriate patient generated tidal volumes despite gradual reduction in pressure support may help to increase a clinician's confidence that a weaning trial will prove successful.

The successful use of pressure support ventilation was recently described for long-term PPV in a puppy with neuromuscular paralysis due to PRN. Following stabilization on a mandatory pressure control mode, the patient was switched to spontaneous pressure support. The authors used the maintenance of adequate tidal volume and ABGs with a gradual reduction and increase in pressure support and flow trigger respectively, as an indication of suitability for weaning (61).

Estimation and planning for patient needs post-extubation is important in optimizing weaning success (52). Ongoing sedation, oxygen and airway requirements should be considered and implemented prior to a weaning attempt. Patients with ongoing laryngeal paralysis may require placement of a TT or CTT (18). Patients with resolving pneumonia should have an oxygen cage prepared or nasal lines placed (52). High flow nasal oxygen is a non-invasive ventilation technique that has been demonstrated to be a safe, effective and well-tolerated means of increasing markers of oxygenation in dyspneic dogs (126). Removal of gastric contents *via* nasogastric tube may reduce the risk of aspiration in this precarious period (116).

## 5. Prognosis

It has been demonstrated that reversible disease processes, including toxicoses (42), left-sided congestive heart failure (54), pulmonary contusions (95), and elapid snake envenomation (127), are associated with higher weaning rates from positive pressure ventilation. Studies examining animals with respiratory failure from TP have also demonstrated favorable outcomes with implementation of MV (30, 118). When financial euthanasia was removed from analysis, Webster et al. reported an overall survival to discharge (STD) of 75% (39/52) in cats and dogs mechanically ventilated for TP (30). When stratified by indication for ventilation, animals treated for ventilatory failure were shown to have a distinct survival benefit [90.5% (19/21) STD] over those treated for hypoxemia [52.6% (10/19)] (30). A 77% (20/26) STD was documented in a more recent analysis of 24 dogs and 2 cats with TP and positive airway cultures from the same institution, suggesting that prognosis may not be influenced by concurrent bronchopneumonia. TP survival rates are higher compared to reported outcomes for veterinary patients undergoing MV for both lower motor neuron disease and all causes, where only 22–23% of animals with pulmonary disease and 21–39% of animals with non-pulmonary disease were discharged from hospital (42, 88, 95).

Two retrospective observational studies documented median duration of MV of 23–24 h (3–152 h), including non-survivors from cardiopulmonary arrest and euthanasia (30, 118). A more recent retrospective analysis reported a longer median duration of MV of 4 days (range 1–10 days) in 26 dogs and two cats with TP and bronchopneumonia (31). Survivors were ventilated for longer (median 5 days, range 2–10 days), which is representative of the confounding effect of euthanasia in earlier literature and supports that TP is a recoverable disease with appropriate intensive care management.

## 6. Areas for future research

MV can be used to successfully manage respiratory failure in cats and dogs with TP, with reported survival rates of 52.6–77 and 90.5% in animals with diseased and healthy lungs, respectively (30, 31). Despite its recognized utility in treating severe TP, there is little published literature which forces clinicians to extrapolate from adjacent human or veterinary MV literature or rely on anecdotal expert recommendation (28). Furthermore, given the narrow geographic distribution of cases, much of the existing data originates from a handful of institutions which may limit its generalizability. Prospective studies investigating and comparing the role of early airway protection, sedation protocols, ventilator modes and settings, lung protective strategies, ARMs, weaning techniques, intravenous fluid therapy, nutrition, and frequency of nursing care on patient outcomes are required to optimize evidence-based management of this condition.

## 7. Conclusion

MV is an important treatment modality in cats and dogs suffering respiratory failure from TP (30, 52, 118) that is indicated when severe hypoxemia, hypercapnia or work of breathing persists in dyspneic patients despite oxygen therapy and sedation (32). The reported median time of MV is 23 h−4 days (3 h−10 days) (30, 31, 118). Ventilator-associated complications include VILI, corneal ulceration and erosions of the tongue (57, 71, 98) which may be exacerbated in animals with TP that have preexisting corneal ulcers, megaesophagus, aspiration pneumonia or laryngeal paralysis causing URT obstruction (1, 15, 19, 30). Further research is required to determine whether ventilation strategies and ancillary care requirements differ for this patient subset when compared to animals ventilated for all causes.

## Author contributions

TO'K and RD performed research, manuscript preparation, and editing. All authors contributed to the article and approved the submitted version.

## References

[1] Atwell R Campbell F Evans E. A prospective survey of tick paralysis in dogs. Aust Vet J. (2001) 79:412–8. 10.1111/j.1751-0813.2001.tb12986.x 11491220

[2] Ross IC. An experimental study of tick paralysis in Australia. Parasitology. (1926) 18:410–29. 10.1017/S0031182000005424

[3] Tibballs J Cooper S. Paralysis with *Ixodes cornuatus* envenomation. Med Jo Aust. (1986) 145:37–8. 10.5694/j.1326-5377.1986.tb113741.x 3724629

[4] Beveridge I Coleman G Gartrell W Hitchman A Whitnall V. Tick paralysis of dogs in Victoria due to *Ixodes cornuatus*. Aust Vet J. (2004) 82:642–3. 10.1111/j.1751-0813.2004.tb12617.x 15887392

[5] Malik R Farrow BRH. Tick paralysis in North America and Australia. Vet Clin North Am Small Anim Pract. (1991) 21:157–71. 10.1016/S0195-5616(91)50016-4 2014620

[6] Cooper B Spence I. Temperature-dependent inhibition of evoked acetylcholine release in tick paralysis. Nature. (1976) 263:693–5. 10.1038/263693a0 185525

[7] Ilklwt JE Turner DM. Infestation in the dog by the paralysis tick, *Ixodes holocyclus* 5. Treatment Aust Vet J. (1988) 65:236–8. 10.1111/j.1751-0813.1988.tb14307.x 3190588

[8] Webster R Mackie J Haskins S. Histopathological changes in the lungs from dogs with tick paralysis: 25 cases (2010-2012). Aust Vet J. (2013) 91:306–11. 10.1111/avj.12085 23889095

[9] Wang Y Watters N Jones E Padula A Leister E Haworth M. Pulmonary histopathology in cats and dogs with fatal tick paralysis. J Comp Pathol. (2022) 197:44–52. 10.1016/j.jcpa.2022.07.005 36089296

[10] Ilkiw J Turner D Howlett C. Infestation in the dog by the paralysis tick *Ixodes holocyclus* 1. Clinical and histological findings. Aust Vet J. (1987) 64:137–9. 10.1111/j.1751-0813.1987.tb09662.x 3632489

[11] Eppleston KR Kelman M Ward MP. Distribution, seasonality and risk factors for tick paralysis in Australian dogs and cats. Vet Parasitol. (2013) 196:460–8. 10.1016/j.vetpar.2013.04.011 23643358

[12] Whitfield Z Kelman M Ward MP. Delineation of an endemic tick paralysis zone in southeastern Australia. Vet Parasitol. (2017) 247:42–8. 10.1016/j.vetpar.2017.09.005 29080763

[13] Goodrich BS Murray MD. Factors influencing the toxicity of salivary gland extracts of *Ixodes holocyclus* Neumann. Int J Parasitol. (1978) 8:313–20. 10.1016/0020-7519(78)90096-6 730466

[14] Holland CT. Asymmetrical focal neurological deficits in dogs and cats with naturally occurring tick paralysis (*Ixodes holocyclus*): 27 cases (1999-2006). Aust Vet J. (2008) 86:377–84. 10.1111/j.1751-0813.2008.00346.x 18826507

[15] Campbell F Atwell R. Megaoesophagus in dogs with tick paralysis (*Ixodes holocyclus*). Aust Vet Pract. (2001) 31:75–9.

[16] Campbell FE Atwell RB. Heart failure in dogs with tick paralysis caused by the Australian paralysis tick, *Ixodes Holocyclus*. Int J Appl Res Vet Med. (2003) 1:148–62.

[17] Leister E Morton J Atwell R Webster R. Clinical presentations, treatments and risk factors for mortality in cats with tick paralysis caused by *Ixodes holocyclus*: 2077 cases (2008–2016). J Feline Med Surg. (2018) 20:465–78. 10.1177/1098612X17733628 28994630 PMC11104066

[18] Hardjo S Nash KJ Day SK Haworth MD. Elective cricothyrotomy in a dog with transient laryngeal paralysis secondary to Australian paralysis tick (*Ixodes holocyclus*) envenomation. Aust Vet J. (2022) 100:440–5. 10.1111/avj.13175 35615962 PMC9546364

[19] Atwell R. Laryngeal paresis caused by *Ixodes holocyclus*. Aust Vet Pract. (2002) 32:41–41.

[20] Ilkiw J Turner D. Infestation in the dog by the paralysis tick *Ixodes holocyclus* 2. Blood-gas and PH, haematological and biochemical findings. Aust Vet J. (1987) 64:139–42. 10.1111/j.1751-0813.1987.tb09663.x 3115241

[21] Ilkiw J Turner D. Infestation in the dog by the paralysis tick *Ixodes holocyclus* 3. Respiratory effects. Aust Vet J. (1987) 64:142–4. 10.1111/j.1751-0813.1987.tb09664.x 3632490

[22] Kim WH Ryu SA. Acute pulmonary edema secondary to upper airway obstruction by bilateral vocal cord paralysis after total thyroidectomy-a case report-. Korean J Anesthesiol. (2012) 62:387. 10.4097/kjae.2012.62.4.387 22558509 PMC3337389

[23] Kitshoff AM van Goethem B Stegen L Vandekerckhov P de Rooster H. Laryngeal paralysis in dogs: an update on recent knowledge. J S Afr Vet Assoc. (2013) 84:E1–9. 10.4102/jsava.v84i1.909 23718178

[24] Schull DN Litster AL Atwell RB. Tick toxicity in cats caused by *Ixodes* species in Australia: a review of published literature. J Feline Med Surg. (2007) 9:487–93. 10.1016/j.jfms.2007.06.006 17706447 PMC10911514

[25] Moses L Harpster N Beck K Hartzband L. Esophageal motility dysfunction in cats: a study of 44 cases. J Am Anim Hosp Assoc. (2000) 36:309–12. 10.5326/15473317-36-4-309 10914528

[26] Blank EL Greenwood B Dodds WJ. Cholinergic control of smooth muscle peristalsis in the cat esophagus. Am J Physiol Gastrointest Liver Physiol. (1989) 257:G517–23. 10.1152/ajpgi.1989.257.4.G517 2572172

[27] Padula AM. Tick paralysis of animals in Australia. In:Gopalakrishnakone P Faiz SMA Gnanathasan CA Habib AG Fernanado R Yang C-C Vogel C-W Tambourgi DV Steifart SA, editors. Clinical Toxinology. Dordrecht: Springer Netherlands (2016), p. 1–20. 10.1007/978-94-007-6288-6_65-1

[28] Atwell R Barker S Barker D Daniel J Holland C Leister E. Tick Paralysis in Dogs and Cats. An Updated Guide to Diagnosis, Management, Treatment and Prevention. North Ryde, NSW: Animal Health Academy (2019).

[29] Ross IC. Tick paralysis in the dog. Aust Vet J. (1934) 10:182–3. 10.1111/j.1751-0813.1934.tb07833.x

[30] Webster R Mills P Morton J. Indications, durations and outcomes of mechanical ventilation in dogs and cats with tick paralysis caused by *Ixodes holocyclus*: 61 cases (2008-2011). Aust Vet J. (2013) 91:233–9. 10.1111/avj.12061 23718792

[31] Tso SSK Leister E Sharp CR Heller J Gibson JS. Positive airway cultures in dogs and cats receiving mechanical ventilation for tick paralysis. Animals. (2022) 12:3304. 10.3390/ani12233304 36496825 PMC9738334

[32] Hopper K Silverstein D editors. Basic mechanical ventilation. In: Small Animal Critical Care Medicine, 2nd ed. St Louis, MO: Saunders Elsevier (2014), p. 161–6. 10.1016/B978-1-4557-0306-7.00030-1

[33] Ilkiw JE Rose RJ Martin ICA. A comparison of simultaneously collected arterial, mixed venous, jugular venous and cephalic venous blood samples in the assessment of blood-gas and acid-base status in the dog. J Vet Intern Med. (1991) 5:294–8. 10.1111/j.1939-1676.1991.tb03136.x 1748981

[34] Farrell KS Hopper K Cagle LA Epstein SE. Evaluation of pulse oximetry as a surrogate for PaO2 in awake dogs breathing room air and anesthetized dogs on mechanical ventilation. J Vet Emerg Crit Care. (2019) 29:622–9. 10.1111/vec.12898 31625687

[35] Neto FJT Carregaro AB Mannarino R Cruz ML Luna SPL. Comparison of a sidestream capnograph and a mainstream capnograph in mechanically ventilated dogs. J Am Vet Med Assoc. (2002) 221:1582–5. 10.2460/javma.2002.221.1582 12479328

[36] Tamura J Itami T Ishizuka T Fukui S Miyoshi K. Central venous blood gas and acid-base status in conscious dogs and cats. J Vet Med Sci. (2015) 77:865–9. 10.1292/jvms.14-0503 25754649 PMC4527512

[37] Maertens B Lin F Chen Y Rello J Lathyris D Blot S. Effectiveness of continuous cuff pressure control in preventing ventilator-associated pneumonia: a systematic review and meta-analysis of randomized controlled trials^*^. Crit Care Med. (2022) 50:1430–9. 10.1097/CCM.0000000000005630 35880890

[38] Aron DN Crowe DT. Upper airway obstruction general principles and selected conditions in the dog and cat. Vet Clin North Am Small Anim Pract. (1985) 15:891–917. 10.1016/S0195-5616(85)50101-1 2416110

[39] Hopper K Powell LL. Basics of mechanical ventilation for dogs and cats. Vet Clin North Am Small Anim Pract. (2013) 43:955–69. 10.1016/j.cvsm.2013.03.009 23747268

[40] Alderson B Senior JM Dugdale AHA. Tracheal necrosis following tracheal intubation in a dog. J Small Anim Pract. (2006) 47:754–6. 10.1111/j.1748-5827.2006.00161.x 17201831

[41] Cagle LA Hopper K Epstein SE. Complications associated with long-term positive-pressure ventilation in dogs and cats: 67 cases. J Vet Emerg Crit Care. (2022) 32:376–85. 10.1111/vec.13177 35001482

[42] Hopper K Haskins S Kass P Rezende ML Aldrich J. Indication, management, and outcome of long-term positive-pressure ventilation in dogs and cats: 148 cases (1900-2001). J Am Vet Med Assoc. (2007) 230:64. 10.2460/javma.230.1.64 17199495

[43] Costerton JW Stewart PS Greenberg EP. Bacterial biofilms: a common cause of persistent infections. Science. (1999) 284:1318–22. 10.1126/science.284.5418.1318 10334980

[44] Hardie EM Spodnick GJ Gilson SD Benson JA Hawkins EC. Tracheal rupture in cats: 16 cases (1983-1998). J Am Vet Med Assoc. (1999) 214:508–12. 10029852

[45] Belson TP. Cuff induced tracheal injury in dogs following prolonged intubation. Laryngoscope. (1983) 93:549–55. 10.1002/lary.1983.93.5.549 6843244

[46] Manabe H Murakami M Kendall A Fulkerson CV. Tracheal stenosis following endotracheal intubation in a dog. Can Vet J. (2021) 62:1289–91. 34857963 PMC8591574

[47] Hung WC Ko JC Weil AB Weng HY. Evaluation of endotracheal tube cuff pressure and the use of three cuff inflation syringe devices in dogs. Front Vet Sci. (2020) 7:39. 10.3389/fvets.2020.00039 32118062 PMC7015870

[48] Epstein S. Care of the ventilator patient. In:Hopper K Silverstein D, editors. Small Animal Critical Care Medicine, 2nd ed. St louis, MO: Saunders Elsevier (2014), p. 185–90. 10.1016/B978-1-4557-0306-7.00034-9

[49] Safdar N Crnich CJ Maki DG. The pathogenesis of ventilator-associated pneumonia: its relevance to developing effective strategies for prevention. Respir Care. (2005) 50:725–39. discussion 739–41. 15913465

[50] Guidelines Guidelines for the management of adults with hospital-acquired ventilator-associated and healthcare-associated pneumonia. Am J Respir Crit Care Med. (2005) 171:388–416. 10.1164/rccm.200405-644ST 15699079

[51] Hedlund CS. Tracheostomies in the management of canine and feline upper respiratory disease. Vet Clin North Am Small Anim Pract. (1994) 24:873–86. 10.1016/S0195-5616(94)50105-0 7817490

[52] Webster RA Haskins S Mackay B. Management of respiratory failure from tick paralysis. Aust Vet J. (2013) 91:499–504. 10.1111/avj.12126 31027398

[53] Nicholson I Baines S. Complications associated with temporary tracheostomy tubes in 42 dogs (1998 to 2007). J Small Anim Pract. (2012) 53:108–14. 10.1111/j.1748-5827.2011.01167.x 22283793

[54] Edwards TH Erickson Coleman A Brainard BM DeFrancesco TC Hansen BD Keene BW. Outcome of positive-pressure ventilation in dogs and cats with congestive heart failure: 16 cases (1992-2012). J Vet Emerg Crit Care. (2014) 24:586–93. 10.1111/vec.12230 25212542

[55] Hopper K. Advanced mechanical ventilation. In:Hopper K Silverstein D, editors. Small Animal Critical Care Medicine, 2nd ed. St Louis, MO: Elsevier Saunders (2014). p. 166–72. 10.1016/B978-1-4557-0306-7.00031-3

[56] Young CC Harris EM Vacchiano C Bodnar S Bukowy B Elliott RRD. Lung-protective ventilation for the surgical patient: international expert panel-based consensus recommendations. Br J Anaesth. (2019) 123:898–913. 10.1016/j.bja.2019.08.017 31587835

[57] Chastre J Fagon JY. Ventilator-associated pneumonia. Am J Respir Crit Care Med. (2002) 165:867–903. 10.1164/ajrccm.165.7.2105078 11934711

[58] Nakayama M Murray PA. Ketamine preserves and propofol potentiates hypoxic pulmonary vasoconstriction compared with the conscious state in chronically instrumented dogs. Anesthesiology. (1999) 91:760–760. 10.1097/00000542-199909000-00029 10485788

[59] Lennon PF Murray PA. Attenuated hypoxic pulmonary vasoconstriction during isoflurane anesthesia is abolished by cyclooxygenase inhibition in chronically instrumented dogs. Anesthesiology. (1996) 84:404–14. 10.1097/00000542-199602000-00020 8602673

[60] Trim C. Anesthetic considerations and complications. In:Paddleford R, editor. Manual of Small Animal Anesthesia, 1st ed. New York, NY: Churchill Livingstone (1999).

[61] Calero Rodriguez A van Oostrom H de Grauw J. Long-term mechanical ventilation of an 8-week-old dog with idiopathic polyradiculoneuritis. Vet Rec Case Rep. (2022) 10:e292. 10.1002/vrc2.292

[62] Boudreau AE Bersenas AME Kerr CL Holowaychuk MK Johnson RJ. A comparison of 3 anesthetic protocols for 24 hours of mechanical ventilation in cats. J Vet Emerg Crit Care. (2012) 22:239–52. 10.1111/j.1476-4431.2012.00722.x 23016813

[63] Ethier MR Mathews KA Valverde A Kerr C Bersenas AM Nykamp SG. Evaluation of the efficacy and safety for use of two sedation and analgesia protocols to facilitate assisted ventilation of healthy dogs. Am J Vet Res. (2008) 69:1351–9. 10.2460/ajvr.69.10.1351 18828695

[64] Mody K Kaur S Mauer EA Gerber LM Greenwald BM Silver G. Benzodiazepines and development of delirium in critically ill children. Crit Care Med. (2018) 46:1486–91. 10.1097/CCM.0000000000003194 29727363 PMC6095819

[65] Authier N Balayssac D Sautereau M Zangarelli A Courty P Somogyi AA. Benzodiazepine dependence: focus on withdrawal syndrome. Ann Pharm Fr. (2009) 67:408–13. 10.1016/j.pharma.2009.07.001 19900604

[66] Walton RAL Enders BD. Suspected benzodiazepine withdrawal-associated seizures in 3 young dogs undergoing mechanical ventilation. J Vet Emerg Crit Care. (2022) 32:800–4. 10.1111/vec.13221 35708738 PMC9796509

[67] Brauer C Jambroszyk M Tipold A. Metabolic and toxic causes of canine seizure disorders: a retrospective study of 96 cases. Vet J. (2011) 187:272–5. 10.1016/j.tvjl.2009.10.023 19939714

[68] Donaldson RE Cortellini S Humm K. Seizure activity following atracurium continuous rate infusion in three mechanically ventilated juvenile dogs. J Vet Emerg Crit Care. (2020) 30:592–6. 10.1111/vec.12989 32780507

[69] Molliex S Dureuil B Montravers P Desmonts JM. Effects of midazolam on respiratory muscles in humans. Anesth Analg. (1993) 77:592–7. 10.1213/00000539-199309000-00029 8368561

[70] Clare M Hopper K. Mechanical ventilation: ventilator settings, patient management, and nursing care. Compend Contin Educ Pract Vet. (2005) 27:256–69.

[71] Pinhu L Whitehead T Evans T Griffiths M. Ventilator-associated lung injury. Lancet. (2003) 361:332–40. 10.1016/S0140-6736(03)12329-X 12559881

[72] Rocco PRM dos Santos C Pelosi P. Pathophysiology of ventilator-associated lung injury. Curr Opin Anaesthesiol. (2012) 25:123–30. 10.1097/ACO.0b013e32834f8c7f 22395439

[73] Ball L Costantino F Orefice G Chandrapatham K Pelosi P. Intraoperative mechanical ventilation: state of the art. Minerva Anestesiol. (2017) 83:1075–1088. 10.23736/S0375-9393.17.11970-X 28528537

[74] Martins ARC Ambrósio AM Fantoni DT Pinto ACBCF Villamizar-Martinez LA Soares JHN. Computed tomography assessment of tidal lung overinflation in domestic cats undergoing pressure-controlled mechanical ventilation during general anesthesia. Front Vet Sci. (2022) 9:842528. 10.3389/fvets.2022.842528 35433898 PMC9011143

[75] Acute Respiratory Distress Syndrome Network Brower RG Matthay MA Morris A Schoenfeld D Thompson BT. Ventilation with lower tidal volumes as compared with traditional tidal volumes for acute lung injury and the acute respiratory distress syndrome. N Engl J Med. (2000) 342:1301–8. 10.1056/NEJM200005043421801 10793162

[76] Slensky K Silverstein D. Mechanical ventilation - advanced concepts. In:Silverstein D Hopper K, editors. Small Animal Critical Care Medicine, 3rd ed. St Louis, MO: Elsevier (2022), p. 192–7.

[77] Higher versus lower positive end-expiratory pressures in patients with the acute respiratory distress syndrome. N Engl J Med. (2004) 351:327–36. 10.1056/NEJMoa032193 15269312

[78] Laffey JG O'Croinin D McLoughlin P Kavanagh BP. Permissive hypercapnia — role in protective lung ventilatory strategies. Intensive Care Med. (2004) 30:347–56. 10.1007/s00134-003-2051-1 14722644

[79] Oura T Rozanski EA Buckley G Bedenice D. Low tidal volume ventilation in healthy dogs. J Vet Emerg Crit Care. (2012) 22:368–71. 10.1111/j.1476-4431.2012.00749.x 22702442

[80] Xincheng L Guanghua G Nianyun W. Effect of lung protective ventilation strategy on oxygenation and pulmonary inflammatory response in dogs with severe smoke inhalation injury. Chin J Integ Tradit West Med Intensive Crit Care. (2015) 6:453–7.

[81] Li W Rosenbruch M Pauluhn J. Effect of PEEP on phosgene-induced lung edema: pilot study on dogs using protective ventilation strategies. Exp Toxicol Pathol. (2015) 67:109–16. 10.1016/j.etp.2014.10.003 25467748

[82] Machado ML Soares JHN Pypendop BH Aguiar AJA Braun C Motta-Ribeiro GC. Cardiovascular and gas exchange effects of individualized positive end-expiratory pressures in cats anesthetized with isoflurane. Front Vet Sci. (2022) 9:865673. 10.3389/fvets.2022.865673 35601404 PMC9114870

[83] de Monte V Grasso S de Marzo C Crovace A Staffieri F. Effects of reduction of inspired oxygen fraction or application of positive end-expiratory pressure after an alveolar recruitment maneuver on respiratory mechanics, gas exchange, and lung aeration in dogs during anesthesia and neuromuscular blockade. Am J Vet Res. (2013) 74:25–33. 10.2460/ajvr.74.1.25 23270342

[84] Ambrósio AM Sanchez AF Pereira MAA Andrade FSRM de Rodrigues RR Vitorasso R de L. Assessment of regional ventilation during recruitment maneuver by electrical impedance tomography in dogs. Front Vet Sci. (2022) 8:815048. 10.3389/fvets.2021.815048 35237676 PMC8882687

[85] Güldner A Kiss T Serpa Neto A Hemmes SNT Canet J Spieth PM. Intraoperative protective mechanical ventilation for prevention of postoperative pulmonary complications. Anesthesiology. (2015) 123:692–713. 10.1097/ALN.0000000000000754 26120769

[86] Canfrán S Gómez de. Segura IA, Cediel R, García-Fernández J. Ef fects of a stepwise lung recruitment manoeuvre and positive end-expiratory pressure on lung compliance and arterial blood oxygenation in healthy dogs. Vet J. (2012) 194:89–93. 10.1016/j.tvjl.2012.02.021 22465618

[87] Fan E Wilcox ME Brower RG Stewart TE Mehta S Lapinsky SE. Recruitment maneuvers for acute lung injury. Am J Respir Crit Care Med. (2008) 178:1156–63. 10.1164/rccm.200802-335OC 18776154

[88] Rutter CR Rozanski EA Sharp CR Powell LL Kent M. Outcome and medical management in dogs with lower motor neuron disease undergoing mechanical ventilation: 14 cases (2003-2009). J Vet Emerg Crit Care. (2011) 21:531–41. 10.1111/j.1476-4431.2011.00669.x 22316200

[89] Esteban A Frutos F Tobin MJ Alía I Solsona JF Valverdu V. A comparison of four methods of weaning patients from mechanical ventilation. N Engl J Med. (1995) 332:345–50. 10.1056/NEJM199502093320601 7823995

[90] Mellema MS Haskins SC. Weaning from mechanical ventilation. Clin Tech Small Anim Pract. (2000) 15:157–64. 10.1053/svms.2000.18293 11109717

[91] Marini J Wheeler A. Critical Care Medicine, 4th ed. Philadelphia, PA: Lippincott Williams & Wilkin (2010), p. 129–47.

[92] Pilbeam S Cairo J. Mechanical Ventilation: Physiological and Clinical Applications, 4th ed. St Louis, MO: Mosby (2006).

[93] MacIntyre N Branson R. Mechanical Ventilation, 2nd ed. Philadelphia, PA: WB Saunders (2009).

[94] Feihl F Perret C. Permissive hypercapnia. How permissive should we be? Am J Respir Crit Care Med. (1994) 150:1722–37. 10.1164/ajrccm.150.6.7952641 7952641

[95] Cagle LA Hopper K Epstein SE. Indications and outcome associated with positive-pressure ventilation in dogs and cats: 127 cases. J Vet Emerg Crit Care. (2022) 32:365–75. 10.1111/vec.13176 35043547

[96] Kallet RH Campbell AR Alonso JA Morabito DJ Mackersie RC. The effects of pressure control versus volume control assisted ventilation on patient work of breathing in acute lung injury and acute respiratory distress syndrome. Respir Care. (2000) 45:1085–96. 10980100

[97] Rittayamai N Katsios CM Beloncle F Friedrich JO Mancebo J Brochard L. Pressure-controlled vs volume-controlled ventilation in acute respiratory failure. Chest. (2015) 148:340–55. 10.1378/chest.14-3169 25927671

[98] Buriko Y Lyons B. Nursing care of the long-term anesthetized patient. In:Creedon JMB Davis H, editors. Advanced Monitoring and Procedures for Small Animal Emergency and Critical Care. Chichester, UK: John Wiley & Sons (2014), p. 589–97. 10.1002/9781118997246.ch46

[99] Chandler JA van der Woerdt A Prittie JE Chang L. Preliminary evaluation of tear production in dogs hospitalized in an intensive care unit. J Vet Emerg Crit Care. (2013) 23:274–9. 10.1111/vec.12055 23647553

[100] Ioannides J Parker J Kumaratunga V Preston J Donaldson D MacFarlane P. A prospective, masked, randomized, controlled superiority study comparing the incidence of corneal injury following general anesthesia in dogs with two methods of corneal protection. Vet Ophthalmol. (2022) 25:291–6. 10.1111/vop.12991 35512022 PMC9541710

[101] Fudge M Anderson JG Aldrich J Haskins SC. Oral lesions associated with orotracheal administered mechanical ventilation in critically III dogs. J Vet Emerg Crit Care. (1997) 7:79–87. 10.1111/j.1476-4431.1997.tb00047.x

[102] Caroff DA Li L Muscedere J Klompas M. Subglottic secretion drainage and objective outcomes. Crit Care Med. (2016) 44:830–40. 10.1097/CCM.0000000000001414 26646454

[103] Nicolson G McGrath A Webster R Li J Kaye S Malik R. NT-proBNP and cardiac troponin I concentrations in dogs with tick paralysis caused by *Ixodes holocyclus*. Aust Vet J. (2016) 94:274–9. 10.1111/avj.12468 27461350

[104] Michard F Teboul JL. Using heart-lung interactions to assess fluid responsiveness during mechanical ventilation. Crit Care. (2000) 4:282. 10.1186/cc710 11094507 PMC137257

[105] Rudloff E. Assessment of hydration. In:Silverstein D Hopper K, editors. Small Animal Critical Care Medicine, 3rd ed. St Louis, MO: Elsevier (2022), p. 372–7.

[106] Saker K Remillard R. Critical care nutrition and enteral-assisted feeding. In:Hand M Thatcher C Remillard R Roudebush P Novotny B, editors. Small Animal Clinical Nutrition, 5th ed. Topeka: Mark Morris Institute (2010), p. 439–76.

[107] Chan D. Nutritional support in the mechanically ventilated small animal patient. In:Chan D, editor. Nutritional Management of Hospitalized Small Animal Patients, 1st ed. West Sussex: John Wiley & Sons (2015), p. 228–32. 10.1002/9781119052951.ch24

[108] Dempsey DT Mullen JL Buzby GP. The link between nutritional status and clinical outcome: can nutritional intervention modify it? Am J Clin Nutr. (1988) 47:352–6. 10.1093/ajcn/47.2.352 3124596

[109] Jolliet P Pichard C Biolo G Chioléro R Grimble G Leverve X. Enteral nutrition in intensive care patients: a practical approach. Intensive Care Med. (1998) 24:848–59. 10.1007/s001340050677 9757932

[110] Greensmith TD Chan DL. Audit of the provision of nutritional support to mechanically ventilated dogs and cats. J Vet Emerg Crit Care. (2021) 31:387–95. 10.1111/vec.13060 33749059

[111] Cereda E Pedrolli C. Recommendations for enteral nutrition: practice is the result of potential benefits, harms, clinical judgment, and ethical issues. J Parenter Enteral Nutr. (2010) 34:103–103. 10.1177/0148607109344626 19861532

[112] Queau Y Larsen JA Kass PH Glucksman GS Fascetti AJ. Factors associated with adverse outcomes during parenteral nutrition administration in dogs and cats. J Vet Intern Med. (2011) 25:446–52. 10.1111/j.1939-1676.2011.0714.x 21457322

[113] Marks SL. The principles and practical application of enteral nutrition. Vet Clin North Am Small Anim Pract. (1998) 28:677–708. 10.1016/S0195-5616(98)50062-9 9597721

[114] Abood S Buffington C. Enteral feeding of dogs and cats: 51 cases (1989-1991). J Am Med Assoc. (1992) 201:619–22. 1517141

[115] van de Putte P Perlas A. Ultrasound assessment of gastric content and volume. Br J Anaesth. (2014) 113:12–22. 10.1093/bja/aeu151 24893784

[116] Crowe DT. Use of a nasogastric tube for gastric and esophageal decompression in the dog and cat. J Am Vet Med Assoc. (1986) 188:1178–82. 3721968

[117] Hopper K. Discontinuing mechanical ventilation. In:Hopper K Silverstein D, editors. Small Animal Critical Care Medicine, 2nd ed. St Louis, MO: Elsevier Saunders (2014), p. 190–4. 10.1016/B978-1-4557-0306-7.00035-0

[118] Trigg NL Leister E Whitney J McAlees TJ. Outcomes of mechanical ventilation in 302 dogs and cats in Australia (2005-2013). Aust Vet Pract. (2014) 44:698–703.

[119] Decramer M Gayan-Ramirez G. Ventilator-induced diaphragmatic dysfunction. Am J Respir Crit Care Med. (2004) 170:1141–2. 10.1164/rccm.2409004 15563638

[120] Chang AT Boots RJ Brown MG Paratz J Hodges PW. Reduced inspiratory muscle endurance following successful weaning from prolonged mechanical ventilation. Chest. (2005) 128:553–9. 10.1016/S0012-3692(15)50395-4 16100137

[121] MacIntyre NR. Evidence-based assessments in the ventilator discontinuation process. Respir Care. (2012) 57:1611–8. 10.4187/respcare.02055 23013898

[122] Esen F Denkel T Telci L Kesecioglu J Tütüncü AS Akpir K. Comparison of pressure support ventilation (PSV) and intermittent mandatory ventilation (IMV) during weaning in patients with acute respiratory failure. Adv Exp Med Biol. (1992) 317:371–6. 10.1007/978-1-4615-3428-0_41 1288147

[123] Brochard L Rauss A Benito S Conti G Mancebo J Rekik N. Comparison of three methods of gradual withdrawal from ventilatory support during weaning from mechanical ventilation. Am J Respir Crit Care Med. (1994) 150:896–903. 10.1164/ajrccm.150.4.7921460 7921460

[124] James RT Miller KS Daniel GL Smith L Reines HD Steven AS. Prospective comparison of IMV and T-piece weaning from mechanical ventilation. Chest. (1989) 96:348–52. 10.1378/chest.96.2.348 2666046

[125] Brochard L Rua F Lorino H Lemaire F Harf A. Inspiratory pressure support compensates for the additional work of breathing caused by the endotracheal tube. Anesthesiology. (1991) 75:739–45. 10.1097/00000542-199111000-00004 1952198

[126] Pouzot-Nevoret C Hocine L Nègre J Goy-Thollot I Barthélemy A Boselli E. Prospective pilot study for evaluation of high-flow oxygen therapy in dyspnoeic dogs: the HOT-DOG study. J Small Anim Pract. (2019) 60:656–62. 10.1111/jsap.13058 31313305

[127] Ong HM Kelers K Hughes D Boller M. Retrospective evaluation of cats with elapid snake envenomation associated neurotoxicity requiring mechanical ventilation: 12 cases (2005-2014). J Vet Emerg Crit Care. (2017) 27:579–85. 10.1111/vec.12632 28799698

